# Wheat and maize allergy: which allergens are involved and relationship with symptoms severity

**DOI:** 10.1186/2045-7022-1-S1-S57

**Published:** 2011-08-12

**Authors:** Elide Anna Pastorello, Giuseppe Scibilia, Laura Farioli

**Affiliations:** 1 Niguarda Ca' Granda Hospital, Allergy and Immunology, Milano, Italy; 2 Niguarda Ca' Granda Hospital, Biochemical Unit, Milano, Italy

## 

Cereals are the basis of human nutrition; they are the food source most intensively produced in the world, surpassing 2000 million tons annually. The production of wheat, corn and rice makes up over the 70% of the total cereal production and thus dominates world agriculture. All 3 cereal crops can determine adverse reactions with different mechanisms of immune-mediated hypersensitivity and through various routes of exposure (digestive, inhalation and contact). A paradigmatic example is that of wheat , which that can cause a range of reactions such as IgE-mediated food allergy, anaphylaxis and asthma, exercise-induced systemic reactions and cell-mediated reactions, such as celiac disease. The major allergens of cereals are represented by alpha-amylase inhibitors, some prolamins, such as gliadins in wheat and zeins in maize, and Lipid Transfer Proteins. These allergens are also differently implicated in the various clinical forms. So that their positivity in many cases actually can be considered diagnostic. The clinical diagnosis of allergy to cereals, because of their nutritional importance, should always be verified by double-blind placebo-controlled food challenged. Long term prognosis is quite favourable and the therapy is based essentially on their elimination, and on the treatment of symptoms in the case of ingestion.

